# Erythrocyte Membrane Docosahexaenoic Acid (DHA) and Lipid Profile in Preterm Infants at Birth and Over the First Month of Life: A Comparative Study with Infants at Term

**DOI:** 10.3390/nu14234956

**Published:** 2022-11-22

**Authors:** Félix Castillo Salinas, Alicia Montaner Ramón, Félix-Joel Castillo Ferrer, Adrià Domingo-Carnice, Begoña Cordobilla, Joan Carles Domingo

**Affiliations:** 1 Service of Neonatology, Hospital Universitari Vall d’Hebron, Universitat Autónoma de Barcelona, E-08035 Barcelona, Spain; 2 Department of Clinical Pharmacology, Hospital Universitari de Bellvitge, L’Hospitalet de Llobregat, E-08907 Barcelona, Spain; 3 Department of Biochemistry and Molecular Biomedicine, Faculty of Biology, Universitat de Barcelona, Avinguda Diagonal 643, E-08028 Barcelona, Spain

**Keywords:** docosahexaenoic acid, arachidonic acid, linoleic acid, eicosapentaenoic acid, lipid profile, preterm infants, erythrocyte membrane

## Abstract

An observational comparative study was designed to assess the fatty acids profile in erythrocyte membrane phospholipids of 30 preterm neonates (<32 weeks gestation) at birth and after 1 month of life versus a convenience sample of 10 infants born at term. The panel of fatty acids included the families and components of saturated fatty acids (SFAs), monounsaturated fatty acids (MUFAs), and n-6 and n-3 polyunsaturated fatty acids (PUFAs) as well as enzyme activity indexes and fatty acids ratios. At birth, the comparison of fatty acid families between preterm and term neonates showed a significantly higher content of SFAs and n-6 PUFAs, and a significantly lower content of MUFAs and n-3 PUFAs in the preterm group. After 30 days of life, significantly higher levels of n-6 PUFAs and significantly lower levels of n-3 PUFAs among preterm neonates persisted. At 30 days of birth, n-6 PUFA/n-3 PUFA and arachidonic acid (ARA) ARA/DHA remained significantly elevated, and DHA sufficiency index significantly decreased in the preterm group. The pattern of n-3 PUFA deficiency at birth and sustained for the first month of life would support the need of milk banking fortified with DHA and the use of DHA supplementation in breastfeeding mothers.

## 1. Introduction

Long-chain polyunsaturated fatty acids (LPUFAs), omega-3 (docosahexaenoic acid [DHA], eicosapentaenoic acid [EPA], and α-linolenic acid [ALA]) and omega-6 (linoleic acid [LA] and arachidonic acid [ARA]) are the major components of brain and retina, and are essential nutrients for growth, development, and function during intrauterine life and after birth [1,2]. The human body is unable to synthesize ALA and LA, so that both omega-3 and omega-6 fatty acids should be obtained from the diet. DHA is derived from ALA by a mechanism of enzymatic biosynthesis, but the extent of conversion of ALA found in components of the diet to DHA appears to be small [3,4]. DHA has shown extensive antioxidant, anti-inflammatory, antiangiogenic, and antiproliferative effects [5,6,7], and more importantly, it plays a crucial role in the pathophysiological mechanisms underlying immune function and neurodevelopment in the newborns [8,9,10]. On the other hand, it has been shown that LPUFAs have pleiotropic effects on cell membranes especially in the regulation of membrane biophysical properties and as lipid mediator precursors and membrane components [11,12,13].

Maturation and development of the nervous system that begins in utero and extends over the first two years of life is a period during which there is an increase in the needs of DHA and ARA of the fetus and newborn [14]. Fetal needs of these LPUFAs are mainly covered by selective transplacental transfer, which increases substantially during the third trimester of pregnancy to satisfy the maximum demands of rapid growth and development of brain neurons, retinal photoreceptor rod cells, and other fetal tissues [15,16]. A shortened gestation and immature enzymatic systems responsible of endogenous synthesis of DHA from chain elongation and desaturation of ALA placed preterm infants in disadvantage compared to term infants regarding LPUFAs status [16,17,18]. Moreover, alterations in LPUFAs of preterm infants at delivery have implications on the risk of early systemic inflammation and diseases associated with prematurity, such as bronchopulmonary dysplasia (BPD), retinopathy of prematurity (ROP), sepsis, or necrotizing enterocolitis (NEC) [19,20,21].

Infants born preterm are especially dependent on an adequate postnatal nutritional supply of DHA, but this is difficult to achieve for different reasons. There is a deficient lipid profile based on analysis of fatty acid composition in food sources for premature infants, plasma or cord blood samples [22,23,24], a need to rely on parenteral nutrition particularly in neonates admitted to the neonatal intensive care unit (NICU), the difficulties of mothers in producing enough breast milk volume for exclusive enteral feeding, and the composition of milk banks based on pooled mature milk with deficient DHA levels. Moreover, the recommended minimal intake of 450 g/day of DHA for pregnant and lactating women is rarely achieved unless there is an increase in DHA intake through fish consumption or nutritional supplements with high-dose DHA while breastfeeding [22]. Interestingly, in a community-based cohort study from South Dakota USA, deficient DHA content in breast milk samples from 84 women who delivered neonates at term was improved by providing nursing mothers with their breast milk DHA level and education about DHA intake while breastfeeding [25]. Regarding an optimal target level for DHA in breast milk, approximately ~0.3% of milk fatty acids has been proposed based on levels achieved in pregnant and lactating women consuming at least 200 mg DHA/day [26].

In a previous study of our group [22], it was shown that the fatty acids profile in samples of donor human milk and mother’s own milk (MOM) at different stages of lactation collected from women who delivered preterm infants had an inadequate content of DHA. Lower levels of DHA in donor milk bank samples were particularly significant as compared with colostrum. Based on these findings, milk banking fortified with DHA would guarantee adequate DHA levels in donor human milk. Following this line of research, this prospective observational study was designed to assess changes in erythrocyte membrane content of fatty acids in premature infants at birth and over the first month of life as compared with infants born at term. Unrecovered low DHA levels in erythrocyte membranes of preterm infants after 1 month of life would further support the need of DHA supplementation in prematurity.

## 2. Materials and Methods

### 2.1. Study Design

This was a prospective observational study conducted at the Service of Neonatology of Hospital Universitari Vall d’Hebron in Barcelona, Spain. The primary objective of the study was to determine the content of fatty acids including saturated fatty acids (SFAs), monounsaturated fatty acids (MUFAs), and n-6 and n-3 PUFAs, in the erythrocyte membrane phospholipids of premature infants born before 32 weeks of gestation, at birth (day 0–1) and at 30 days of life, in comparison with values at birth of infants at term. The secondary objective was to determine changes in the erythrocyte membrane content of fatty acids in the group preterm infants over the course of the first month of life.

The study protocol was approved by the Clinical Research Ethics Committee of Hospital Universitari Vall d’Hebron (code PRM(AMI)287/2017, approval date 11 August 2017). Written informed consent was obtained from parents or legal representatives of all infants who participated in the study within 24 h after birth of their neonates.

### 2.2. Participants and Samples

Participants were preterm neonates born alive before 32 weeks of gestation from women of any race or parity attended at our hospital at the time of delivery, who required admission to the Service of Neonatology for neonatal care between January and December 2018. Preterm infants with life-threatening congenital malformations were excluded as were those for whom the written informed consent was not obtained. To be included in the group of preterm term infants, it was required to have available blood samples collected at different time intervals (at birth and at 1 and 2 days, half week, 1 week, 1 and a half week, 2 weeks, and 1 month of life). Between February and March 2021, a convenience sample of neonates born at term after 37 weeks of gestation was selected. To be included in the group of term neonates, it was required to have available a blood sample collected at birth (day 0–1). The signed informed consent from the parents or legal guardians was required for all preterm and term neonates. In all cases, blood sampling for laboratory analyses that included a complete blood cell count was obtained under routine clinical conditions and standard elective or urgent indications for laboratory testing, and in no case blood samples were drawn as the only justification for the purpose of the study.

Nutrition guidelines at the Service of Neonatology in all premature infants born before 32 weeks of pregnancy and/or birth weight < 1500 g included enteral feeding during the first 24 h of life (if feasible according to the infant’s clinical condition) and complementary parenteral nutrition to ensure nutritional requirements. The standard parenteral nutrition composition for the first day of life in infants weighing < 1250 g and in those weighing between 1250 and 1500 g is as follows: fluids 80 and 70 mL/kg/day, carbohydrates 7 and 6.1 g/kg/day, amino acids 2.4 and 2.1 g/kg/day, SMOflip (1%) 0.8 and 0.6 g/kg/day, calcium 1.6 and 1.4 mEq/kg/day, phosphorus 0.86 and 0.75 mmol/kg/day, and sodium 1.6 and 1.4 mEq/kg/day, respectively.

### 2.3. Analysis of Fatty Acids

Blood samples (1 mL) were obtained from each infant by venipuncture in tubes with anticoagulation (EDTA or citrate). After removal of plasma by centrifugation, samples were stored at −80 °C until analysis. The composition of fatty acids in red blood cells (RBC) was determined as methyl esters after a methylation reaction using the method of Lepage and Roy [27]. Samples were transferred to glass tubes for direct transesterification. Methanol-hexane 4:1 (*v*/*v*) was added with an internal standard and 0.01% butylhydroxytoluene to prevent oxidation of fatty acids. Then, acetyl chloride was added and the tubes were tightly closed with Teflon-lined caps, gasified with nitrogen, and shaken for 30 s.

Fatty acids were analyzed by gas chromatography (GC) using a gas chromatograph mass spectrometer (GCMS-QP2010 Plus, Shimadzu, Kyoto, Japan), Shimadzu AOC-20i auto injector and Shimadzu AOC-20 autosampler. A Suprawax-280 high polarity capillary column (Teknokroma Analítica, S.A., Barcelona, Spain), 15 m × 0.10 mm internal diameter, 0.10 µm film thickness was used. Data were acquired by GCMS solution software. Functioning conditions were optimized to analyze the whole spectrum of fatty acids. Mass spectrometry operating parameters included scan rate 10,000 amu/s, mass range of 40–400 m/z, and capillary voltage 1.0 kV. The interface and ion source temperatures were set at 255 °C and 200 °C, respectively. The peaks of fatty acid methyl esters (FAMEs) were identified through electron ionization mass spectra using NIST11 library and through GC retention times, comparing with a reference FAME mixture (GLC-744, Nu-Che Prep. Inc., Elysian, MN, USA). The results were expressed in relative amounts (percentage molar of total fatty acids) of duplicate sampling.

### 2.4. Panel of Fatty Acids

The panel of fatty acids analyzed included the following: saturated fatty acids (SFAs) (myristic acid C14:0, palmitic acid C16:0, stearic acid C18:0, arachidic acid C20:0, behenic acid C22:0, and lignoceric acid C24:0); monounsaturated fatty acids (MUFAs) (palmitoleic acid C16:1 n7, oleic acid C18:1 n9, cis-vaccenic acid C18:1 n7, gondoic acid C20:1 n9, erucic acid C22:1 n9, and nervonic acid (C24:1 n9); n-6 PUFAs (linoleic acid [LA] C18:2 n6), ɣ-linoleic acid C18:3 n6, cis-11,14 eicosadienoic acid C20:2 n6, dihomo-ɣ-linolenic acid [DHGLA] C20:3 n6, ARA C20:4 n6, adrenic acid C22:4 n6, and osbond acid or docosapentaenoic acid [DPA n6] C22:5 n6); and n-3 PUFAs (ALA C18:3 n3, eicosapentaenoic acid [EPA] C20:5 n3, docosapentaenoic acid [DPA n3] C22:5 n3, and DHA C22:6 n3).

In addition, other fatty acid ratios were calculated, including omega-3 index (EPA+DHA), n-6 PUFA/n-3 PUFA, ARA/DHA, ARA/EPA, EPA/DHA, LA/ARA, anti-inflammatory fatty acid index (AIFAI) ((DHGLA+EPA+DHA)/ARA), essential fatty acids index (EFASTI) ([Σn-3+Σn-6]/[Σn-7+Σn-9]), DHA sufficiency index (DHASI) (22:6 n3 [DHA]/docosapentaenoic acid [DPA n6] 22:5 n6), DHA deficiency index (DHADI) (DPA n6/adrenergic acid C22:4 n6).

On the other hand, other enzyme activity indexes were calculated, including stearoyl-CoA desaturase-16 (16-SCD) (C16:1 n7/C16:0), stearoyl-CoA desaturase-18 (18-SDC) (C18:1 n9/C18:0), delta-5-desaturase (D5D) (ARA/DGLA), delta-6-desaturase (D6D) (DGLA/LA), and elongase (C18:0/C16:0).

### 2.5. Statistical Analysis

Categorical variables are expressed as frequencies and percentages and continuous variables as mean and standard deviation (SD) or median and range. Differences in fatty acid composition of the erythrocyte membrane between preterm and term infants were analyzed using the Student’s *t* test for independent samples. All comparisons were made in reference to data obtained at birth in neonates at term. Statistical significance was set at *p* < 0.05. The the GraphPad Prism program, version 9.00 for Windows (GraphPad Software, San Diego, CA, USA, www.graphpad.com) was used for the analysis of data.

## 3. Results

### 3.1. Characteristics of the Study Population

A total of 65 preterm neonates born at less than 32 weeks of pregnancy who were admitted to the Service of Neonatology during the study period were eligible. The group of neonates at term included a convenience sample of 10 infants born at a gestational age of 37 weeks or greater. Infant and maternal characteristics of both preterm and term groups are shown in Table 1. Maternal data were very similar, with a mean age of 32.6 (6.4) years in the preterm group and 31.8 (4.2) in the term group. Countries of origin were also similar with most women being from Spain.

These 65 preterm neonates met the eligibility criteria of gestational age of less than 32 weeks of pregnancy and admission to the Neonatology Service during the study period, but 35 (53.8%) were excluded mostly because of logistic reasons related to unavailability of adequate blood samples or inability to obtain the written informed consent within 24 h of life. Therefore, 30 preterm neonates were included in the study, 17 of which (56.7%) had blood samples available at 30 days of life. In the remaining 13 preterm neonates, blood samples at 30 days were not available mainly due to difficulties in adequacy of sample collection and storage. In relation to SMOflip, infants received a median (minimum-maximum) of 2 (1–2.5) mL parenteral fats for a median of 7.4 (2–10 days).

### 3.2. Fatty Acid Composition of Erythrocyte Membrane

At birth, the comparison of fatty acid families between preterm and term neonates showed a significantly higher content of SFAs and n-6 PUFAs, and a significantly lower content of MUFAs and n-3 PUFAs in the preterm group. After 30 days of life, significantly higher levels of n-6 PUFAs and significantly lower levels of n-3 PUFAs among preterm neonates persisted (Table 2).

The analysis of changes of individual compounds of the fatty acid families is shown in Table 3. In relation to SFAs, there were significantly higher erythrocyte membrane levels of stearic acid (C18:0) (*p* = 0.036) and lignoceric acid (C24:0) (*p* < 0.0001) in preterm neonates at birth as compared with infants at term, but after 30 days of life, differences disappeared. Among MUFAs, significantly lower values of oleic acid (C18:1 n9) in preterm neonates at birth (*p* < 0.0001), also improved at 30 days of life, being similar to those of infants at term at the time of birth. Differences in nervonic acid (C24:1 n9) were not found.

In the group of n-6 PUFAs, preterm babies at birth showed significantly lower levels of LA (C18:2 n6) (*p* < 0.0001) and significantly higher levels of ARA (C20:4 n6) (*p* < 0.0001) as compared with neonates at term, but higher ARA levels (*p* = 0.017) remained at 30 days, whereas LA levels increased up to a similar levels than those observed in term neonates at birth. In the group of n-3 PUFAs, levels of ALA (C18:3 n3), EPA (C20:5 n3), and DPA n3 (C22:5 n3) at birth were significantly lower (*p.* < 0.0001) than those of neonates at term, and at 30 days, DPA n3 levels still remained significantly lower (*p* = 0.0001). The levels of DHA were somewhat higher at birth in the preterm group, but after 30 months remained lower than in neonates at tem, although differences were not statistically significant.

**Figure 1:**
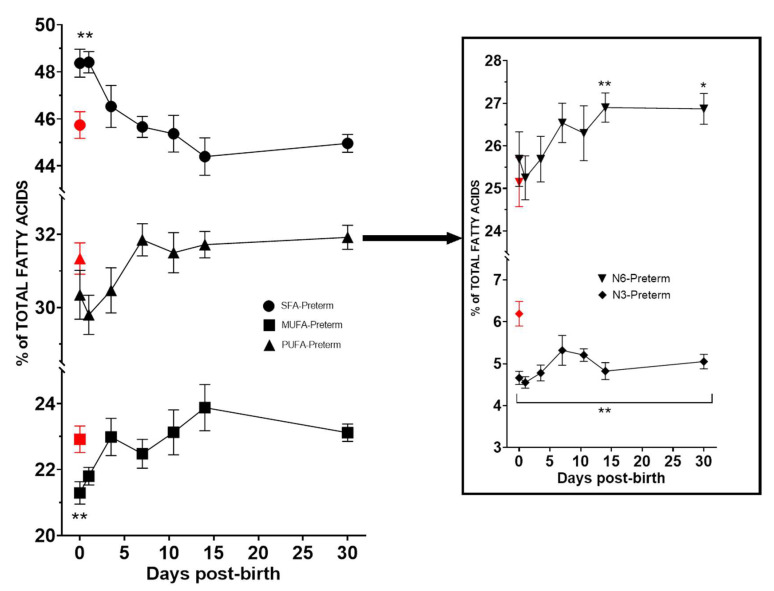
Evolution of the main fatty acid families in preterm neonates during the first 30 days of life as compared with values at birth on neonates at term (red symbol) (asterisks indicate statistically significant differences, *p* < 0.05, * *p* < 0.05, ** *p* < 0.001).

The evolution of erythrocyte membrane content of SFAs, MUFAs, and PUFAs in the group of preterm neonates during the first month of life, and particularly n-6 and n-3 PUFAs is shown in Figure 1. The erythrocyte membrane content of n-3 PUFAs remained significantly (*p* < 0.05) below the level found at birth in term neonates during the first 30 days of life. By contrast, the erythrocyte membrane content of n-6 PUFAs was significantly higher than values at birth of neonates at term from day 15 (Figure 1).

### 3.3. Fatty Acid Rations and Enzyme Activity Indexes

At birth, there were statistically significant differences between preterm and term neonates in almost all fatty acid ratios, in particular higher values of n-6 PUFA/n-3 PUFA, ARA/DHA, and ARA/EPA, and lower values of EPA/DHA, LA/ARA, DHASI, DHADI. At 30 days of birth, n-6 PUFA/n-3 PUFA and ARA/DHA remained significantly elevated, and omega-3 index and DHASI significantly decreased in the preterm group (Table 4).

**Figure 2:**
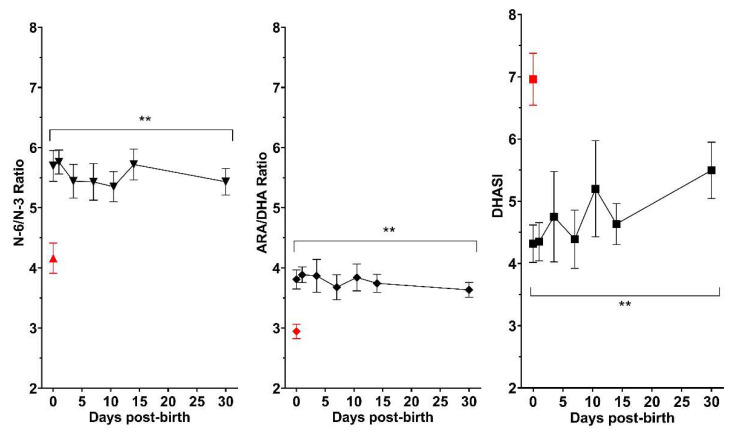
Evolution of fatty acids ratios in preterm neonates during the first 30 days of life as compared to mean values in term neonates at birth (red symbol). N-6/n-3 PUFAs ratio and ARA/DHA remained higher, whereas lower DHASI values persisted (ARA: arachidonic acid, DHA: docosahexaenoic acid; DHASI: DHA sufficiency index. (asterisks indicate statistically significant differences, *p* < 0.05).

In relation to the evolution of main fatty acid ratios in preterm neonates, n6-PUFA/n-3 PUFA, ARA/DHA, and DHASI remained significantly different during the entire period analyzed as compared with values in neonates born at term (Figure 2).

## 4. Discussion

This study analyzed a complete lipid profile of fatty acids of red blood cell membrane in preterm neonates at birth and during the first month of life as compared with neonates at term. Other studies have evaluated erythrocyte membrane fatty acids in preterm infants, but the panel of fatty acids was limited to the overall composition of total SFAs, MUFAs, PUFAs (n-6 and n-3) and some individual compounds (e.g., LA, ARA, DHA, n-6/n-3 PUFA or ARA/DHA ratios) [21,28,29]. In this respect, the main novelty of the study is the evaluation of the principal components of fatty acids families together with a panel of enzyme activity indexes and ratios in a group of preterm neonates followed over the first month of life. Also, a comparison was made of erythrocyte membrane levels of fatty acids between preterm and term neonates at birth and at 1 month of age, although considering the mean gestational age of 28.7 months, most preterm neonates at 1 month would have a corrected gestational age of 32 weeks. However, the accelerated maturation during the first month of life brings preterm neonates closer to infants born at term at 37 weeks of gestation.

We found that preterm neonates at birth showed higher levels of SFAs and n-6 PUFAs than babies born at term, together with lower levels of MUFAs and n-3 PUFAs. These differences, however, in SFAs and MUFAs normalized after 1 month of life. Interestingly, the higher levels of n-6 PUFAs at birth corresponded to greater values of ARA and a reduction of LA, which increased to reach normal values at 1 month, whereas ARA remained significantly high.

On the other hand, an important finding of the study was a reduced level of n-3 PUFAs that persisted after 1 month of life in relation to low levels of ALA, EPA, and DPA at birth. After 1 month of life, ALA and EPA levels were similar to those of neonates at term, but lower levels of DPA and DHA persisted. At 1 month of life, a decreased level of DHA, with an insufficient increase in the EPA, are consistent with a concomitant decrease in omega-3 index. This is a very important aspect for DHA supplementation during the critical period of the first weeks of life, which would contribute to increase DHA levels at the age of 1 month. Optimal DHA levels may be related with a decrease of retinopathy and inflammatory events, such as bronchopulmonary dysplasia, although both conditions are multifactorial but having prematurity as a common event.

The analysis of the ratios of n-6 PUFA/n-3 PUFA, ARA/DHA, ARA/EPA, and the percentage of AIFAI at birth in preterm neonates may suggest a pro-inflammatory status in preterm neonates. However, proinflammatory proteins were not measured. Sustained inflammation in association with fragile immune defense mechanism is regarded as a crucial mediator for mortality and the development of morbidities in preterm infants [30,31]. Also, the balance between metabolites of n-3 and n-6 PUFAs seems to be important in pregnancy maintenance and have a significant role in cervical dilatation and initiation of labour [32]. In randomized, double-blind clinical trials carried out in preterm neonates, the administration of medium-chain triglyceride/n-3 PUFA-enriched intravenous fat emulsion changed the fatty acid profile consistent with an attenuated inflammatory response [33,34].

Preterm neonates’ changes in fatty acids profile at month of life also confirm the maintenance of the pro-inflammatory status since the n-6 PUFA/n-3 PUFA ratio continues persisting significantly higher as compared with term neonates at birth as well as high values of other biomarkers, such as ARA/DHA, ARA/EPA, and AFAI. In addition, whereas the ARA to DHA ratio is initially high in the fetus, the transfer of DHA increases during the last trimester of gestation, resulting in a lower ARA to DHA ratio at term. In a cohort study of 175 infants with a median gestational age of 25 weeks, higher mean daily serum levels of DHA during the first 28 postnatal days were associated with less severe ROP even after adjustment for known risk factors, but only in infants with sufficiently high ARA levels [21].

Independently of significantly lower values of n-3 PUFAs in preterm neonates at birth and at 1 month of age, a remarkable finding of the study was the maintenance of decreased levels of the DHA sufficiency index (DHASI), which further indicates the lack of amelioration of DHA levels through the first 30 days of life. Although LPUFAs synthesis occurs in preterm infants, the endogenous synthesized LPUFAs are insufficient to meet requirements defined by fetal accretion rates, and there is extensive evidence of the importance of adequate DHA levels for normal development of the infant brain and vision system and to prevent other potential long-lasting detrimental neurodevelopmental effects that extend beyond the period of DHA insufficiency [35,36]. In a systematic review and meta-analysis of data reported in 38 trials of the effects of n-3 PUFA supplementation on child cognitive and visual outcomes up to the first 2 years of life, it was found that supplementation with DHA+EPA+ARA or DHA+ARA in preterm infants (7 trials) improved Bayley mental developmental index (MDI) and visual acuity [37].

Other studies have evaluated the values of fatty acids in phospholipids of red blood cell membranes of term and preterm infants. In a study of 37 term infants at 3 days of life, infants fed with a formula in which high-DHA/low-EPA fish oil was added to levels similar to that in human milk up to 4 months of age showed signicantly higher concentrations of DHA than the standard formula-fed group [29]. In this study, however, the use of this supplement was associated with a decrease of ARA. In very low birth weight infants, supplementation of formulas with n-3 and n-6 PUFAs in amounts typical for human milk fat resulted in similar fatty acids profiles in plasma and erythrocyte membrane phospholipids as found during breast milk feeding [38]. Other studies have consistently demonstrated the need to add n-3 PUFAs for preterm infant formulas to obtain fatty acid profiles comparable to infants receiving human milk [39,40]. In a previous study of our group, donor human milk had an inadequate content of DHA for feeding preterm infants, so that milk banking fortified with DHA would ensure adequate DHA levels [22]. Also, DHA supply dose in preterm infants dependently increased plasma DHA levels [41,42]. However, Smith and Rouse [43] published a comprehensive review of DHA and the preterm infant and have drawn attention to inconsistencies of data reported in the literature regarding the optimal dosage and method of delivery of DHA, as well as to the need of further studies to optimize DHA intake in preterm neonates.

The small number of preterm neonates followed over the first month of life is the main limitation of the study. Differences in the recruitment period between preterm and term neonates may be explained by the COVID-19 pandemic, but the characteristics related to maternal age and countries of origin were similar in the two groups and they probably represent the same underlying population because of the single-center nature of the study. On the other hand, the limited number of 10 infants born at term may be explained by logistic difficulties associated with: (a) ethical reasons to drawn a blood sample under routine medical care of healthy full-term neonates in the lack of an indication for laboratory testing; (b) difficulties to obtain the informed consent at 0–1 days when blood sampling was taken; (c) logistic difficulties in storage of samples and transfer to the laboratory for analysis. However, the extensive lipid profile panel analyzed reinforces the study findings. Correlations between fatty acids composition of phospholipids of red blood cells and enzymatic activity indexes with maternal age, gestational age, or birth weight may be interesting lines of research for futures studies.

The relationship between the lipid profile pattern in red blood cells and the clinical morbidity of prematurity was not analyzed in our study. A recent systematic review and meta-analysis of four randomized controlled trials with 1966 neonates suggests that DHA supplementation was not associated with a lower incidence of bronchopulmonary dysplasia at 36 weeks of postmenopausal age among preterm infants, and does not seem to exert significant clinical benefits on other outcomes, such as NEC, severe ROP, intraventricular hemorrhage or sepsis [44]. Nevertheless, the authors stated that the certainty of evidence classified by the GRADE approach was low in this meta-analysis and a follow-up research may be needed to clarify these results [44].

Finally, the impact of SMOF lipid emulsions in the feeding of preterm infants on the levels of DHA and ARA merits a comment. In the present study, analyzing the contribution of SMOF lipid to the group of preterm infants with an average birth weight of 1098 g was a maximum of 2.5 mL during the first 7.4 days of life. This is equivalent to a maximum daily intake of 15 mg/kg/day of DHA and 1.6 mg/kg/day of ARA. These intakes are much lower than maternal intakes during the third trimester which are considered to be around 60 mg/kg/day of DHA [45,46]. Therefore, possible variations in DHA and ARA levels contributed by SMOF lipid feeding do not appear to be relevant to the observed changes. Moreover, it has been reported that the use of SMOF lipid emulsions in early preterm infants did not prevent the decline in DHA relative to birth levels [47,48]. However, in contrast to our results, studies of early preterm infants showed lower levels of ARA in the plasma phospholipids of infants receiving SMOF emulsions compared to infants receiving soybean oil [48,49].

## 5. Conclusions

In the present study of the erythrocyte membrane lipid profile in preterm neonates at birth as compared with neonates at term, a main finding was the pattern of n-3 PUFA deficiency at birth and sustained for the first month of life. Although during the postnatal period especially in preterm infants, the importance of an adequate supply of ARA cannot be minimized, the present findings would support the need of milk banking fortified with DHA, as well as DHA supplementation to neonates when feeding with mother’s own milk only until the first corrected gestational month.

## Figures and Tables

**Table 1:** Infant and maternal characteristics of preterm neonates and infants at term.

Variables	Neonates	
	Preterm (*n* = 65)	Term (*n* = 10)
Infant characteristics		
Gestational age, weeks, mean (SD) (range)	28.7 (2.2) (24.4–32.3)	38.5 (1.7) (37.2–41)
Birth weight, g, mean (SD) (range)	1098 (338.7) (470–1910)	2798 (354.6) (2170–4050)
Male sex, *n* (%)	37 (59.6)	6 (60)
Cesarean section, *n* (%)	51 (78.5)	2 (20)
Maternal characteristics		
Age, years, mean (SD) (range)	32.6 (6.4) (17–48)	31.8 (4.2) (22–39)
Gravida, median (range)	1 (0–14)	2 (0–3)
Country of origin, *n* (%)		
Spain	45 (69.2)	7 (70)
Other European countries	5 (7.7)	0
South America	3 (4.6)	0
North Africa	4 (6.2)	0
Sub-Saharan Africa	1 (1.5)	2 (20)
Asia	7 (10.8)	1 (10)
Nutritional management		
Age starting enteral feeding, hours, mean (SD)	22.5 (6–144)	
Age at full enteral feeding, days, mean (SD)	9.8 (5–18)	
Total parenteral nutrition, *n* (%)	64 (98.5)	
Days of total parenteral nutrition, mean (SD)	7.4 (0–36)	

SD: standard deviation.

**Table 2:** Content of fatty acid families in erythrocyte membranes at birth and at 30 days of life in preterm neonates as compared with infants born at tem.

Fatty Acid Family (% Molar of Total Fatty Acid)	Term Infants at Birth (*n* = 10)	Preterm Infants at Birth (*n* = 30)	*p* Value	Preterm Infants at 30 Days (*n* = 17)	*p* Value
SFAs	45.74 (1.79)	48.37 (3.25)	**0.003**	44.96 (1.55)	0.365
MUFAs	22.92 (1.27)	21.29 (1.86)	**0.005**	23.12 (1.09)	0.421
PUFAs	31.34 (1.35)	30.35 (3.66)	0.938	31.92 (1.36)	0.333
n-6 PUFAs	24.06 (1.19)	25.69 (3.50)	**0.004**	26.87 (1.50)	**<0.0001**
n-3 PUFAs	6.19 (0.92)	4.66 (0.86)	**<0.0001**	5.05 (0.71)	**0.001**

Data as mean and standard deviation (SD) in parenthesis. SFAs: saturated fatty acids; MUFAs: monounsaturated fatty acids; PUFAs: polyunsaturated fatty acids; n-6: omega-6; n-3: omega-3.

**Table 3:** Content of individual fatty acids of the main families in erythrocyte membranes at birth and at 30 days of life in preterm neonates as compared with infants born at term.

Fatty Acid (% Molar of Total Fatty Acid)	Term Infants at Birth (*n* = 10)	Preterm Infants at Birth (*n* = 30)	*p* Value	Preterm Infants at 30 Days (*n* = 17)	*p* Value
SFAs					
Palmitic acid (C16:0)	23.53 (1.59)	23.16 (1.86)	0.114	22.13 (1.49)	0.079
Stearic acid (C18:0)	16.16 (1.54)	17.09 (0.90)	**0.036**	15.95 (0.70)	0.702
Lignoceric acid (C24:0)	3.57 (0.38)	5.43 (0.88)	**<0.0001**	4.03 (0.86)	0.114
MUFAs					
Oleic acid (C18:1 n9)	14.52 (1.09)	12.05 (1.44)	**<0.0001**	14.60 (0.87)	0.666
Nervonic acid (C24:1 n9)	5.38 (0.90)	5.44 (0.88)	0.275	5.30 (0.53)	0.980
n-6 PUFAs					
LA (C18:2 n6)	9.05 (1.22)	3.91 (0.71)	**<0.0001**	8.60 (1.88)	0.242
ARA (C20:4 n6)	11.30 (1.24)	15.35 (2.32)	**<0.0001**	12.79 (1,53)	**0.017**
n-3 PUFAs					
ALA (C18:3 n3)	0.13 (0.04)	0.06 (0.04)	**<0.0001**	0.11 (0.05)	0.216
EPA (C20:5 n3)	0.88 (0.62)	0.16 (0.08)	**<0.0001**	0.58 (0.36)	0.407
DPA (C22:5 n3)	1.31 (0.33)	0.30 (0.09)	**<0.0001**	0.80 (0.27)	**0.0002**
DHA (C22:6 n3)	3.86 (0.34)	4.15 (0.79)	0.236	3.56 (0.47)	0.076

Data as mean and standard deviation (SD) in parenthesis. SFAs: saturated fatty acids; MUFAs: monounsaturated fatty acids; PUFAS: polyunsaturated fatty acids; n-6: omega-6; LA: linoleic acid; ARA: arachidonic acid; n-3: omega-3; ALA: α-linolenic acid; EPA: eicosapentaenoic acid; DPA: docosapentaenoic acid; DHA: docosahexaenoic acid.

**Table 4:** Estimated fatty acid ratios and enzyme activity indexes.

	Term Infants at Birth (*n* = 10)	Preterm Infants at Birth (*n* = 30)	*p* Value	Preterm Infants at 30 Days (*n* = 17)	*p* Value
Fatty acid ratios:					
Omega-3 index	4.73 (0.60)	4.30 (0.81)	0.150	4.14 (0.53)	**0.015**
n-6 PUFA/n-3 PUFA	4.16 (0.80)	5.70 (1.40)	**0.0003**	5.43 (0.91)	**0.0018**
ARA/DHA	2.90 (0.38)	3.81 (0.87)	**0.0003**	3.63 (0.51)	**0.0006**
ARA/EPA	25.34 (23.46)	132.10 (91.86)	**<0.0001**	36.06 (22.85)	0.242
EPA/DHA	0.23 (0.17)	0.038 (0.020)	**<0.0001**	0.18 (0.18)	0.464
LA/ARA	0.81 (0.17)	0.26 (0.07)	**<0.0001**	0.70 (0.25)	0.053
AIFAI, %	57.38 (11.62)	44.02 (8.71)	**<0.0001**	49.54 (7.50)	**0.028**
EFASTI	1.37 (0.10)	1.44 (0.25)	0.739	1.38 (0.10)	0.814
DHASI	8.70 (2.51)	4.32 (1.65)	**<0.0001**	5.50 (1.87)	**0.001**
DHADI	0.28 (0.07)	0.38 (0.06)	**0.0006**	0.28 (0.07)	0.719
Enzyme activity indexes:					
16-SCD	0.037 (0.024)	0.034 (0.010)	0.453	0.025 (0.011)	0.177
18-SCD	0.88 (0.16)	0.71 (0.10)	**0.0019**	0.92 (0.07)	0.173
D5D	6.88 (1.41)	6.97 (1.87)	>0.999	5.93 (1.20)	**0.023**
D6D	0.18 (0.10)	0.60 (0.13)	**<0.0001**	0.27 (0.08)	**0.001**
Elongase	0.69 (0.10)	0.74 (0.14)	0.120	0.72 (0.05)	0.143

Data as mean and standard deviation (SD) in parenthesis. PUFA: polyunsaturated fatty acid; ARA: arachidonic acid; DHA: docosahexaenoic acid; EPA: eicosapentaenoic acid; LA: linoleic acid; AIFAI: anti-inflammatory fatty acid index; EFASTI: essential fatty acid index; DHASI: DHA sufficiency index; DHADI: DHA deficiency index; SCD: stearoyl-CoA desaturase; D5D: delta-5-desaturase; D6D: delta-6-desaturase (D6D).

## Data Availability

Data are available from the authors (F.C.S. and J.C.D.) upon request.
